# Evaluation of the 2b-RAD method for genomic selection in scallop breeding

**DOI:** 10.1038/srep19244

**Published:** 2016-01-12

**Authors:** Jinzhuang Dou, Xue Li, Qiang Fu, Wenqian Jiao, Yangping Li, Tianqi Li, Yangfan Wang, Xiaoli Hu, Shi Wang, Zhenmin Bao

**Affiliations:** 1 Ministry of Education Key Laboratory of Marine Genetics and Breeding, College of Marine Life Sciences, Ocean University of China, China; 2 Department of Computational and Systems Biology, Genome Institute of Singapore, Singapore; 3 Laboratory for Marine Fisheries and Aquaculture, Qingdao National Laboratory for Marine Science and Technology, China; 4 Laboratory for Marine Biology and Biotechnology, Qingdao National Laboratory for Marine Science and Technology, China

## Abstract

The recently developed 2b-restriction site-associated DNA (2b-RAD) sequencing method provides a cost-effective and flexible genotyping platform for aquaculture species lacking sufficient genomic resources. Here, we evaluated the performance of this method in the genomic selection (GS) of Yesso scallop (*Patinopecten yessoensis*) through simulation and real data analyses using six statistical models. Our simulation analysis revealed that the prediction accuracies obtained using the 2b-RAD markers were slightly lower than those obtained using all polymorphic loci in the genome. Furthermore, a small subset of markers obtained from a reduced tag representation (RTR) library presented comparable performance to that obtained using all markers, making RTR be an attractive approach for GS purpose. Six GS models exhibited variable performance in prediction accuracy depending on the scenarios (e.g., heritability, sample size, population structure), but Bayes-alphabet and BLUP-based models generally outperformed other models. Finally, we performed the evaluation using an empirical dataset composed of 349 Yesso scallops that were derived from five families. The prediction accuracy for this empirical dataset could reach 0.4 based on optimal GS models. In summary, the genotyping flexibility and cost-effectiveness make 2b-RAD be an ideal genotyping platform for genomic selection in aquaculture breeding programs.

Genomic selection (GS), which was initially proposed by Meuwissen *et al.*^1^, can greatly increase the genetic gain and reduce the generation interval through the selection of candidates based on the genomic estimated breeding values (GEBVs) calculated using genome-wide single-nucleotide polymorphisms (SNPs). After the successful implementation of GS in dairy cattle^2^, its applicability has also been investigated in maize, wheat and apple breeding programs^3^,^4^,^5^. In the field of aquaculture breeding, previous studies have focused on investigating the benefits of implementing this approach in family-based breeding schemes using simulations^6^,^7^,^8^, and demonstrate that higher-accuracy breeding values could be obtained by GS compared with the traditional breeding method. A recent empirical study conducted in Atlantic salmon using an admixed population also confirmed the advantage of GS in aquaculture breeding^9^.

Nonetheless, one major premise of the use of GS in practical breeding is the requirement of sufficient genetic markers. High-density markers ensure that the linkages between markers and quantitative trait loci (QTLs) are tight so that recombination does not cause them to decay rapidly^1^, and therefore QTLs can be determined by the neighboring markers. Traditionally, it is difficult for aquacultural breeders to obtain high-density markers at a low cost and this situation is even worse for species with little or no genomic resources. Recent development of genotyping-by-sequencing (GBS) methods that reduce genome complexity via restriction enzymes^10^,^11^, is revolutionizing the way of genetic marker discovery and genotyping. The most significant advantage of GBS methods for the implementation of GS in breeding programs is the low per-sample cost needed to generate tens of thousands of molecular markers. It is notable that 2b-restriction site-associated DNA (2b-RAD) represents one of efficient GBS methods, which features even and tunable genome coverage and provides a flexible genotyping platform to meet diverse research purposes^12^,^13^,^14^,^15^,^16^. The prediction accuracy achieved using GBS data is comparable to that obtained using the SNP array datasets in the recent maize breeding project^17^. Currently, it remains largely unknown whether the marker density provided by GBS methods is sufficient for accurately estimating breeding values for aquaculture breeding, although these methods have significant advantages in reducing the cost of high-density marker genotyping.

Yesso scallop (*Patinopecten yessoensis*), which is cultured mainly in Liaoning and Shandong provinces of China, has been among the most important commercial shellfish since its introduction from Japan in the early 1980 s^18^. Conventional breeding approaches, such as polyploidy breeding^19^, species hybridization^20^, and gynogenesis^21^ have been investigated for genetic improvement of Yesso scallop. Most recently, extensive transcriptomic resources have been generated for Yesso scallop^22^,^23^,^24^,^25^, and a number of growth- and immune-related genes have been characterized^26^,^27^,^28^,^29^,^30^. A whole-genome sequencing project for this species has been initiated by our group (NCBI BioProject no. PRJNA259405), making it an ideal subject for GS.

The objective of this study was to evaluate the potential applicability of the 2b-RAD method in the GS of Yesso scallop through simulation and empirical data analyses. Key factors affecting prediction accuracy of breeding values were assessed, such as marker density, heritability, and statistical models. Our study supports 2b-RAD to be a very powerful and promising tool for genomic selection in aquaculture breeding programs.

## Results

### Simulation data analysis

We first investigated whether the marker densities generated by the 2b-RAD method are sufficient to capture the QTL effects using GS models. We generated high-density marker panels (HD-SNPs) consisting of 250 k SNPs spaced evenly along the Yesso scallop genome, medium-density marker panels (MD-SNPs) consisting of 61 k SNPs located in BsaXI tags (i.e., 5′-N_10_ACN_5_CTCCN_8_-3′), and low-density marker panels (LD-SNPs) consisting of 5 k SNPs located in reduced tag representation (RTR)-BsaXI tags (i.e., 5′-AN_9_ACN_5_CTCCN_7_T-3′), which contain approximately 250, 50, and 5 markers per Mb, respectively. Among all SNPs, 5,000 loci were randomly chosen as candidate QTLs with the allelic effects sampled from a normal distribution with a mean of 0 and a variance of 1. The initial breeding population was simulated for 1,000 generations according to the Fisher-Wright population model with the genetic parameter values specified in Table 1 (see Simulation 1). The genomic prediction results in G_1_ for three panels with different statistical models are summarized in Table 2. Small difference was observed between HD-SNPs and MD/LD-SNPs for each statistical model under different heritabilities, suggesting that the marker densities generated by 2b-RAD exhibited comparable performance with that obtained using the array-based genotyping technology. Notably, at heritability values greater than 0.2, prediction accuracies obtained with LD-SNPs using G-BLUP, BayesA, and BayesB models are similar to those obtained with MD-SNPs. For example, the accuracy of BayesA can reach 0.92 with a heritability of 0.5 using only LD-SNPs. In terms of the statistical models, G-BLUP, RR-BLUP, BayesA, and BayesB exhibited the highest prediction accuracy for all cases with no significant differences among them. In addition, the advantage of these models over LASSO and BL was more pronounced at heritability values lower than 0.2. One possible explanation for the poorer performance of shrinkage and selection approaches (LASSO and BL) is that the genetic variance is largely uniformly distributed over all of the chromosomes specified in our simulation dataset. It is well known that minimizing the cost function by variable selection could result in an upward bias of the estimates of marker effects^31^. The over-generation prediction accuracies (G_1_ − > G_2_) are provided in Table 2, with no significant difference observed among the three marker-density panels, which is likely due to high LD within families.

Given the reliance of many aquaculture breeding schemes on sib testing^6^, we further investigated the impact of sample size on genomic prediction for a family-based breeding population under low marker density (Table 1, Simulation 2). The breeding population composed of 20 families with each containing 50 individuals. To create datasets with different sample sizes, 5, 10, 15 and 20 families were randomly chosen and combined, resulting in population sizes ranging from 250 to 1000. To enable a uniform comparison with the empirical data analysis in the following section, only a subset of 2b-RAD markers (2,364) randomly chosen from the LD-SNP panel were utilized. Principal coordinate analysis (PCA) and genetic kinship analysis suggested that most of the 20 families can be genetically separated (Supplementary Figs S1a,b). Table 3 shows the prediction accuracies for different levels of family combinations using six GS models. At a heritability greater than 0.2, the prediction accuracies using only 5 families can range from 0.83 to 0.92 for G-BLUP, BayesA and BayesB, and no significant improvement was obtained with the sample size increasing up to 1,000 (20 families). At a low level of heritability (i.e., *h*^2^ = 0.1), a substantial increase in accuracy was observed with the inclusion of more individuals in the training set. For example, the prediction accuracy for BayesB increased by 12% with a sample size of 500 (10 families), and by 14% with a sample size of 1,000 (20 families), indicating that sample size and phenotype heritability should be considered simultaneously in the implementation of GS. Overall, even for a dataset consisting of 250 individuals (5 families), acceptable prediction accuracies (over 0.8) could be obtained by selection of optimal statistical models (e.g. G-BLUP and Bayes-alphabet).

### Real data analysis

**Figure 1:**
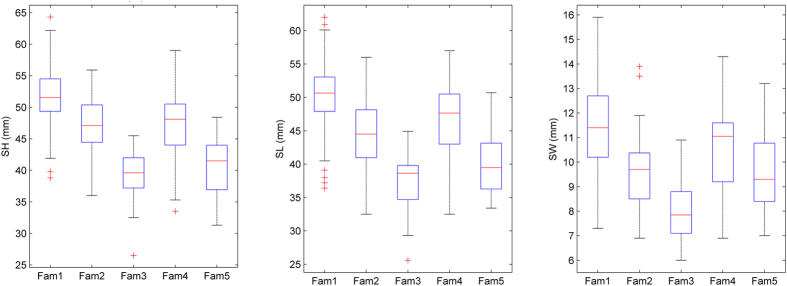
Box and whisker plots of three traits shown for five Yesso scallop families. SH, shell height; SL, shell length; SW, shell width.

**Figure 2:**
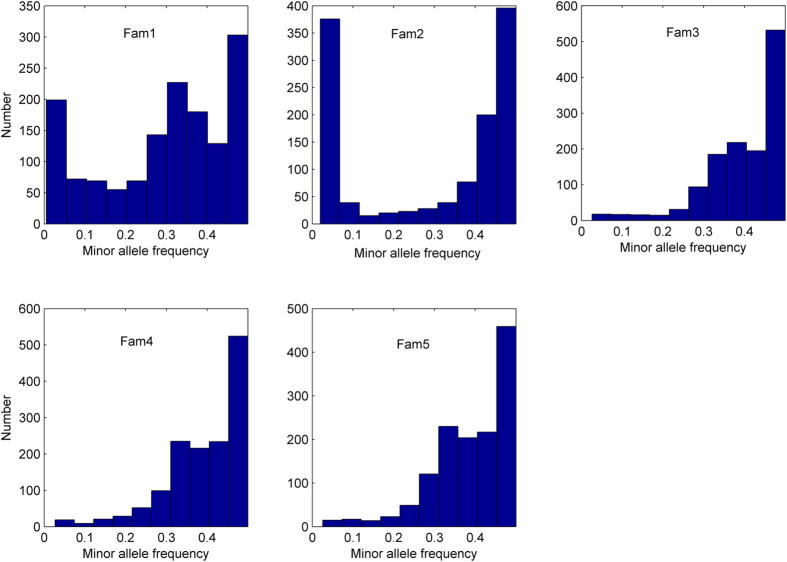
Distribution of the minor allele frequencies of 2,364 markers in five Yesso scallop families.

**Figure 3:**
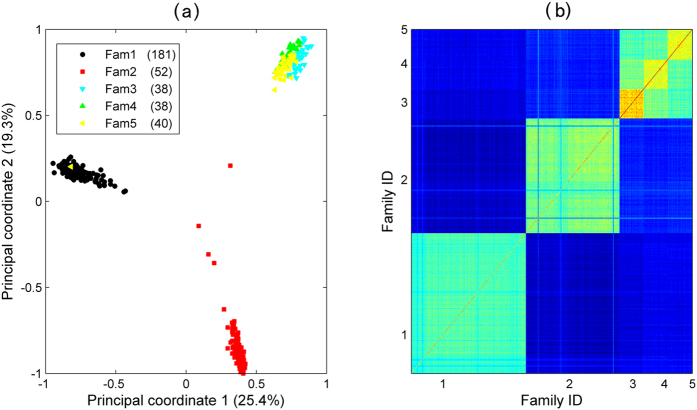
Principal component analysis (a) and genetic kinships (b) of the five empirical families based on 2,364 markers.

The real dataset was composed of 349 Yesso scallop individuals that were derived from two full-sib families and three bi-parental families. Box and whisker plots exhibited the first and third quartiles of shell length (SL), shell width (SW), and shell height (SH) among the five families (Fig. 1). According to the one-way ANOVA analysis, the *p* values among the five families for SL, SW, and SH were statistically significant, with values of 2e-6, 2e-6 and 3.6e-3, respectively. For all individuals, 2b-RAD reads and mapping rates were summarized in Supplementary Table S1. After screening for minor allele frequency (>5%) and SNP calling frequency (>70%), a high-quality set of SNPs (2,364) with an average calling rate of 84% was used in genomic selection models (Fig. 2). PCA and genetic kinship analysis suggested that the three bi-parental families were closer to each other but are genetically distinct from the other two full-sib families (Fig. 3a,b). Significant genotypic variance estimates had been observed among all these traits using the entire population, with medium heritabilities (0.36 ~ 0.48). Meanwhile, for single families, the heritability ranged from 0.28 to 0.61 for SH, from 0.26 to 0.60 for SL, and from 0.15 to 0.48 for SW (Table 4).

**Figure 4:**
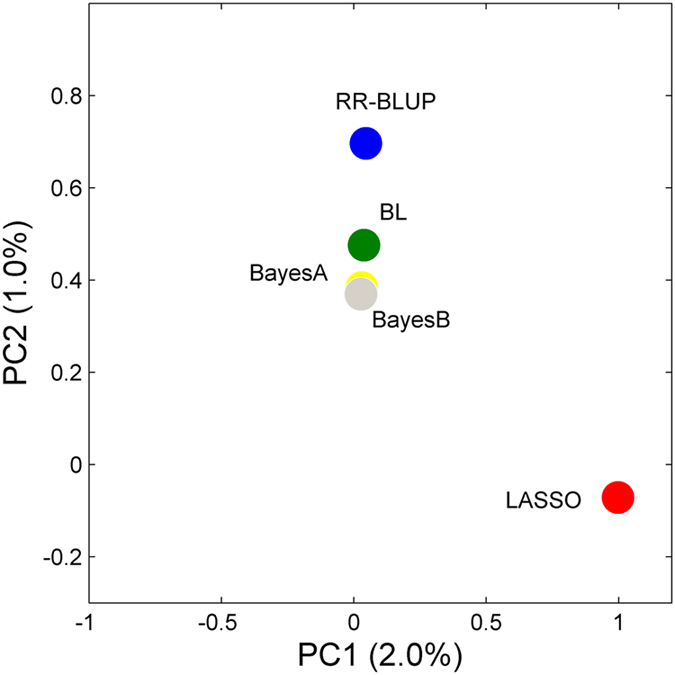
Principal component analysis of five GS models based on the estimated genetic effects of 2,364 markers. G-BLUP is not included in comparison because genetic effect is not estimated for individual markers in this model.

The prediction accuracies assessed using five-fold cross-validation for the entire population are shown in Table 5. The prediction accuracies varied from 0.15 to 0.40 across the three traits, which were substantially lower than those obtained from the family-based simulation analysis (Table 3). This difference can be partly attributed to the fact that the prediction accuracy for the real dataset was calculated based on the correlation between the observed phenotypes and GEBVs, as the true breeding values is unknown in practice. By dividing the square-root of the corresponding heritability, the adjusted accuracies could reach 0.6 across these methods, which is still lower than that obtained in the simulation case. The coefficient of regression (slope) of the observed phenotype on the estimated breeding values was calculated as a measurement of the bias of each method. For all situations, the slopes of these models were not significantly different from 1.0, with the largest deviation being less than 0.06, indicating the absence of significant bias in the prediction. G-BLUP, BayesA and BayesB outperformed the other methods due to their better performance across the three traits (Table 5). The genetic effects of all markers that were calculated based on five GS models were shown in Supplementary Table S2. The PCA analysis based on all marker effects demonstrated that LASSO is markedly different from the other methods (Fig. 4), as was also confirmed by pairwise comparisons among these methods with the pair of LASSO and BayesB having the largest derivation for the SL trait (Table 6).

## Discussion

### 2b-RAD: a cost-effective genotyping platform for genomic selection

The comprehensive set of restriction-site associated sequences generated by the 2b-RAD method provides an excellent fractional representation of the targeted genome^12^,^13^,^14^,^15^,^16^. The expected number of polymorphic markers can be readily predicted based on the total number of restriction sites and the polymorphism rate in a given genome. For Yesso scallop, approximately 242,044 BsaXI sites were identified from the reference genome dataset (~0.97G, unpublished), generating approximately 61,000 SNPs at a polymorphism rate of 2% (i.e., MD-SNPs). The prediction accuracies obtained by using MD-SNPs were comparable to those obtained by using HD-SNPs (Table 2), indicating the feasibility of determining an optimal sequencing plan that balances prediction accuracy and sequencing cost. This finding is also in agreement with the results of a recent empirical investigation in an Atlantic salmon breeding project which revealed that increasing the SNP density to over 22k had no substantial improvement on the genomic accuracy^9^. The generality of this observation, however, needs to be investigated in more aquaculture species, as marker density needed for GS implementation is also dependent on other factors, such as population structure, mating schemes, effective population size and mutation rate. For species with large genomes, sequencing all BsaXI sites at a depth of 20x for all individuals remains a substantial investment. For example, sequencing 1,000 Yesso scallop individuals would require approximately 5 billion reads, which are approximately equivalent to the number of reads produced from >30 sequencing lanes using the HiSeq2000 platform. Of course, high genome prediction (>90%) under this situation can be obtained (Table 2). A notable feature of the 2b-RAD method is the tunable genome representation from RTR libraries that are constructed using less degenerate adaptors^12^,^14^. For example, only 1/10^th^ of total BsaXI sites in the Yesso scallop genome are targeted by using adaptors with 5′-NNA-3′ overhangs. Thus, the sequencing cost can dramatically decrease compared with that cost associated with the use of a standard BsaXI library, and the prediction abilities in this case remain acceptable (Tables 3 and 5). Our empirical data analysis suggests that integrating multiple families in a training set can be regarded as an effective approach to GS, not only because the effects of markers can be estimated from a relatively larger number of phenotypes but also low-density markers may be sufficient to pick up high linkage disequilibrium within full-sib families.

### Comparison of the simulated and empirical cases

There is a decline of prediction accuracy from the simulation case to the empirical case even when both cases have a similar sample size and a similar marker density. Potential reasons for this decrease in accuracy include but are not limited to the following: (i) The genetic architecture. It is challenging to generate a simulated dataset that mostly resembles to a real case because genetic backgrounds of real breeding families/populations are usually unavailable in practice. Most of existing GS models do not consider non-additive effects and may partly misclassify the non-additive effects into the random error term, resulting in a decrease of the additive heritability. To explore this possibility, we performed an additional simulation analysis (Table 1, Simulation 3), considering the dominant effects, one source of non-additive genetic effects. When the dominant and additive variances relative to the total genetic variance are both 0.3, the prediction accuracies for Bayes-alphabet approaches will decrease by approximately 20% (Supplementary Fig. S2) in comparison with the additive-only simulation datasets (Table 3), but prediction difference between the new simulation and real datasets becomes smaller. Although a model that includes both additive and non-additive genetic effects could be beneficial for exploitation of specific combining ability^32^,^33^, the computational demand for these models is generally high and usually requires greater computing resources or more efficient algorithms. (ii) The sample size. Although our simulation analysis suggested that small family-based sample size could achieve reasonable prediction accuracies (Table 2), it does not necessarily apply to all types of real datasets which can be substantially different from simulation datasets. Hence, a careful examination should be performed before drawing inferences for situations in practical aquaculture breeding. (iii) The markers closest to a QTL may not be segregating in breeding families. For example, we observed a higher number of monogenic markers in the families 3, 4 and 5 in contrast to the other families (data not shown), which may cause some QTL regions to be undetected due to the lack of segregating markers. (iv) Multiple QTL alleles. SNP markers are usually biallelic and can thus only distinguish two alleles. If multiple alleles at a QTL are present and the QTL is adjacent to a SNP, it is quite possible that one SNP allele may be linked to more than one QTL allele. Therefore, the presence of identical SNP alleles in different samples does not necessarily imply identical QTL alleles.

### Comparison of different GS models

In this study, we evaluated a wide range of GS models for their potential use in aquacultural GS projects. It is currently challenging to find a statistical model that is optimal for all breeding projects, as each model has its advantages and disadvantages depending on the scenarios (heritability, sample size, population structure, etc.)^31^. As expected, different performance was observed among these models under both simulation and empirical analyses. The Bayes-alphabet and BLUP-based models had relatively better performance in all cases than the other models because they can effectively capture the polygenic resemblance and genetic relationships^31^,^34^,^35^.

## Conclusion

Our simulation and empirical analyses support 2b-RAD to be a powerful and cost-effective genotyping platform for GS implementation in aquaculture breeding programs. Comparison of six GS models revealed variable performance in prediction accuracy depending on the scenarios (e.g., heritability, sample size, population structure), but Bayes-alphabet and BLUP-based models generally outperformed other models though additional, larger studies are required to verify these suggestive findings.

## Methods

### Genetic resource simulation

The simulation dataset for *in silico* analysis was created from the draft genome sequence of Yesso scallop (~0.97G, unpublished). We first introduced SNPs at a rate of 2% by adding alleles to the diploidized genome. Hence, approximately 5,000 loci were randomly chosen as candidate QTLs and the allelic effects were sampled from a normal distribution with a mean of 0 and a variance of 1. The 2b-RAD method was then used for *in silico* marker genotyping. Two marker panels with different marker densities were generated by extracting all BsaXI tags (i.e., 5′-N_10_ACN_5_CTCCN_8_-3′) and RTR-BsaXI tags (i.e., 5′-AN_9_ACN_5_CTCCN_7_T-3′) generated using selective adaptors with the 5′-NNA-3′ overhangs. Only SNPs located in the BsaXI tags were considered for subsequent simulation analysis.

### Breeding population simulation

A breeding population was simulated for 1000 generations according to the Fisher-Wright population model using the quantiNemo software^36^. The detailed parameters for the generation of simulated populations/families can be found in Table 1. The first simulation was generated as follows: 100 male and 100 female candidates from G_1000_ were selected as sires and dams and randomly mated in pairs with 20 offspring/pair to generate G_1_. This process was repeated to generate G_2._ For all of the samples in G_1_ and G_2_, the markers were genotyped and the traits were recorded. Phenotypic records were generated by adding the genetic values to a normally distributed error term and the variance was determined by heritability. The second simulation was composed of 20 full-sib families with each one containing 50 offspring by mating 10 males and 10 females from G_1000_ randomly. Different from the second experiment, the third simulation considered not only the additive genetic effects, but also dominant effects.

### 2b-RAD experiments and data analysis

A total of 349 Yesso scallop individuals that were derived from two full-sib families and three biparental families were included in 2b-RAD sequencing and genotyping. These families were established with assistance of the Dalian Zhangzidao Fishery Group Corporation. Growth-related traits including shell length (SL, mm), shell width (SW, mm), and shell height (SH, mm) were measured for all samples at the age of 15 months. The 2b-RAD libraries were prepared following the protocol developed by Wang *et al.*^12^ and were subject to single-end sequencing using an Illumina HiSeq2000 platform. 2b-RAD genotyping was performed using the RADtyping program v1.5^37^ with default parameters. Segregating markers that could be genotyped in at least 70% of the individuals with minor allele frequency (>5%) were retained for subsequent analysis. Missing genotype values were estimated using the mean algorithm implemented in the R package rrBLUP^38^. In addition, associations among the genotypes were analyzed by principal component analysis (PCA) using the MATLAB software. Estimates of the narrow-sense heritability (*h*^*2*^) of each trait were obtained as the ratio of additive variance (σ_a_^2^) to the total phenotypic variance (σ_a_^2^ + σ_e_^2^) using the REML algorithm^38^ with the genetic relationship matrix calculated using 2,364 genetic markers.

### Cross-validation

To validate the accuracy of family/population-based prediction, we divided all of the samples into five subsets. Four of the subsets (80%) were used to estimate the marker effects, whereas the remaining subset (20%) was used as the validation set. For the over-generation prediction, all of the samples in G_1_ were considered as the training set, and the samples in G_2_ were used as the validation set. The prediction accuracy *r* was calculated using the correlation between the true breeding values (TBVs) and the estimated breeding values (EBVs) by sampling the training and validation sets for 100 times. For the empirical data analysis, the prediction accuracy *r* was adjusted according to the Equation (1) because the true breeding values are unknown in practice.


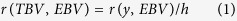


where *y* is the observed phenotype and *h* is the square-root of heritability.

### Statistical models

Six GS models including G-BLUP, BayesA, BayesB, Random Regression Best Linear Unbiased Prediction (RR-BLUP), Least Absolute Shrinkage And Selection Operator (LASSO), and Bayesian LASSO (BL) were used to estimate the marker effects. The basic model is as follows:


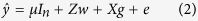


where *y* is the vector of the phenotype for a given trait, *μ* is an intercept, *Z* is a design matrix assigning individuals to families, *w* is the vector of the family effect, and *X* is a design matrix allocating records to the SNP effects, in which element *X*_*ij*_ = 0, 1, or 2 if the genotype of individual *i* at the *j*^th^ SNP is *AA*, *AB*, or *BB*, respectively.

### BLUP

For the traditional BLUP method, the genetic effect is defined as following:


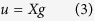


It follows that


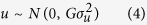


Where *G* is the kinship coefficient matrix determined by the pedigree information.

### G-BLUP

Different from the traditional BLUP method, G-BLUP estimates the kinship coefficient matrix based on the genome-wide genotyping information.


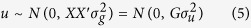


where *G* = *XX’k* with a common choice of *k* as follows:


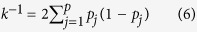


where *p*_j_ is an estimate of the frequency of the allele codes at the *j*th marker. Therefore, the representation of G-BLUP is given by the following model:


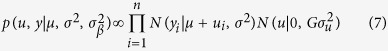


The posterior mode of this approach can be rewritten as:


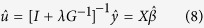


In other words, the method can be understood easily by replacing the standard pedigree-based numerator relationship matrix used in the traditional BLUP approach with a marker-based estimate of additive relationships.

### BayesA

For the BayesA approach, the following prior assumption regarding the distribution of SNP effects made:


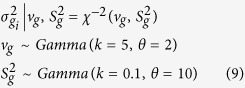


### BayesB

For BayesB, a priori SNP effect is assumed to be zero with probability *π*_g_, and normally distributed with a mean equal to 0 and a locus-specific variance with probability (1–*π*_g_). BayesA is a special case of BayesB in which *π*_g_ = 0.


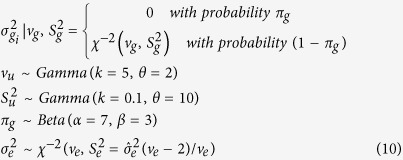


### RR-BLUP

For RR-BLUP, a vector of SNP effects *g* is assumed to be normally distributed, and the direct solution of equation (2) would be obtained:


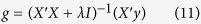


where λ = σ_e_^2^/(σ_g_^2^/*k*), *k* = 2*p*_i_(1-*p*_i_) and *p*_i_ is the allelic frequency of the *i*^th^ marker.

### BL

For the BL approach, *g* is assigned a prior double exponential (*DE*) distribution:


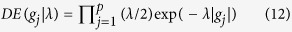


And the residual variance σ_e_^2^ is assigned a scaled inverse chi-square prior distribution.

### LASSO

For the LASSO approach, the genetic effect *g* is a solution to an optimization problem of the following form


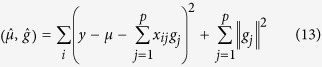


The RR-BLUP method was implemented using the rrBLUP package^38^, whereas the LASSO approach was implemented using the glmnet package^39^, and the others were implemented using the BLR packages^40^. The breeding values for the validation population were estimated as:


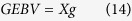


## Additional Information

**How to cite this article**: Dou, J. *et al.* Evaluation of the 2b-RAD method for genomic selection in scallop breeding. *Sci. Rep.* **6**, 19244; doi: 10.1038/srep19244 (2016).

## Supplementary Material

Supplementary Figures S1 and S2

Supplementary Table S1

Supplementary Table S2

## Figures and Tables

**Table 1:** The parameters used for scallop population/family simulation.

Simulation	Dataset type	Sample size	Marker density	Genetic model
1	Population-based	2,000	HD-SNPs (250 k) MD-SNPs (61 k) LD-SNPs (5 k)	Additive
2	Family-based	20 × 50^a^	LD-SNPs (2,364)	Additive
3	Family-based	5 × 50	LD-SNPs (2,364)	Additive + Dominant

^a^20 × 50 denotes a population composed of 20 sub-families with each containing 50 samples.

**Table 2:** Accuracy of GEBVs estimated from the simulated population-based datasets (Simulation 1) under different heritabilities.

Case	Marker density	Method	*h*^*2*^ = 0.1	*h*^*2*^ = 0.2	*h*^*2*^ = 0.3	*h*^*2*^ = 0.4	*h*^*2*^ = 0.5
G_1_		BLUP	0.29	0.47	0.53	0.68	0.70
	HD	LASSO	0.50	0.54	0.65	0.79	0.78
		RR-BLUP	0.74	0.82	0.89	0.92	0.92
		BL	0.29	0.42	0.47	0.63	0.65
		G-BLUP	0.74	0.81	0.88	0.94	0.94
		BayesA	0.74	0.82	0.88	0.94	0.94
		BayesB	0.73	0.82	0.89	0.94	0.93
	MD	LASSO	0.39	0.56	0.60	0.75	0.78
		RR-BLUP	0.69	0.86	0.87	0.92	0.92
		BL	0.23	0.39	0.54	0.63	0.65
		G-BLUP	0.70	0.86	0.87	0.92	0.92
		BayesA	0.70	0.86	0.87	0.92	0.92
		BayesB	0.70	0.86	0.87	0.92	0.92
	LD	LASSO	0.44	0.50	0.64	0.77	0.83
		RR-BLUP	0.47	0.72	0.88	0.90	0.92
		BL	0.23	0.33	0.43	0.55	0.66
		G-BLUP	0.69	0.82	0.84	0.90	0.92
		BayesA	0.72	0.82	0.86	0.90	0.92
		BayesB	0.76	0.82	0.90	0.90	0.92
G_1_- > G_2_		BLUP	0.24	0.37	0.57	0.59	0.76
	HD	LASSO	0.39	0.62	0.61	0.68	0.81
		RR-BLUP	0.77	0.90	0.91	0.91	0.94
		BL	0.59	0.76	0.79	0.82	0.89
		G-BLUP	0.74	0.87	0.92	0.93	0.94
		BayesA	0.73	0.86	0.91	0.93	0.93
		BayesB	0.73	0.87	0.91	0.93	0.95
	MD	LASSO	0.31	0.48	0.63	0.77	0.78
		RR-BLUP	0.73	0.85	0.91	0.94	0.94
		BL	0.26	0.39	0.48	0.59	0.65
		G-BLUP	0.72	0.85	0.91	0.93	0.93
		BayesA	0.73	0.85	0.91	0.93	0.93
		BayesB	0.73	0.86	0.91	0.93	0.93
	LD	LASSO	0.44	0.67	0.72	0.83	0.87
		RR-BLUP	0.27	0.89	0.89	0.92	0.93
		BL	0.31	0.38	0.51	0.56	0.65
		G-BLUP	0.76	0.88	0.88	0.92	0.94
		BayesA	0.80	0.85	0.89	0.92	0.94
		BayesB	0.81	0.89	0.89	0.92	0.93

**Table 3:** Accuracy of GEBVs estimated from the simulated family-based datasets (Simulation 2) under the low marker density.

No. of families	Model	*h*^*2*^ = 0.1	*h*^*2*^ = 0.2	*h*^*2*^ = 0.3	*h*^*2*^ = 0.4	*h*^*2*^ = 0.5
5	BLUP	0.27	0.39	0.47	0.63	0.69
	LASSO	0.33	0.43	0.41	0.52	0.60
	RR-BLUP	0.27	0.28	0.32	0.32	0.42
	BL	0.16	0.24	0.39	0.56	0.58
	G-BLUP	0.63	0.84	0.89	0.91	0.92
	BayesA	0.67	0.83	0.89	0.90	0.92
	BayesB	0.66	0.83	0.90	0.90	0.92
10	BLUP	0.31	0.43	0.55	0.63	0.69
	LASSO	0.24	0.45	0.49	0.60	0.69
	RR-BLUP	0.24	0.28	0.70	0.61	0.66
	BL	0.14	0.39	0.41	0.53	0.65
	G-BLUP	0.74	0.82	0.84	0.90	0.93
	BayesA	0.79	0.81	0.84	0.90	0.93
	BayesB	0.82	0.84	0.87	0.90	0.92
15	BLUP	0.36	0.44	0.55	0.63	0.69
	LASSO	0.25	0.48	0.61	0.72	0.77
	RR-BLUP	0.25	0.28	0.88	0.88	0.92
	BL	0.13	0.37	0.43	0.54	0.68
	G-BLUP	0.66	0.83	0.89	0.88	0.93
	BayesA	0.68	0.83	0.89	0.89	0.93
	BayesB	0.68	0.84	0.89	0.90	0.93
20	BLUP	0.38	0.44	0.57	0.67	0.70
	LASSO	0.37	0.50	0.65	0.75	0.79
	RR-BLUP	0.31	0.84	0.87	0.90	0.92
	BL	0.25	0.35	0.50	0.57	0.61
	G-BLUP	0.70	0.75	0.86	0.90	0.92
	BayesA	0.75	0.80	0.86	0.90	0.92
	BayesB	0.80	0.84	0.87	0.91	0.92

**Table 4:** Estimation of variance components and heritabilities for three traits including shell height (SH), shell length (SL) and shell width (SW).

	Across-family	Fam1	Fam2	Fam3	Fam4	Fam5
SH						
σ_a_^2^	14.58 (2.56^a^)	9.21 (2.49)	6.38 (2.13)	8.56 (1.10)	12.27 (4.21)	17.8 (2.75)
σ_e_^2^	15.84 (1.18)	14.88 (1.05)	16.31 (1.15)	7.01 (0.55)	19.09 (1.51)	14.2 (0.96)
h^2^	0.48 (0.05)	0.38 (0.07)	0.28 (0.08)	0.41 (0.05)	0.39 (0.09)	0.54 (0.06)
SL						
σ_a_^2^	16.30 (2.68)	9.93 (3.07)	7.24 (3.11)	1.79 (0.87)	7.74 (1.73)	5.92 (2.51)
σ_e_^2^	17.48 (1.16)	16.42 (1.32)	20.47 (1.39)	14.23 (0.98)	25.81 (2.14)	15.29 (1.34)
h^2^	0.48 (0.05)	0.38 (0.08)	0.26 (0.06)	0.11 (0.05)	0.23 (0.05)	0.26 (0.09)
SW						
σ_a_^2^	2.66 (0.29)	0.52 (0.37)	2.15 (0.35)	0.91 (0.04)	2.53 (0.14)	1.91 (0.16)
σ_e_^2^	4.68 (0.15)	3.07 (0.15)	2.37 (0.15)	1.24 (0.04)	1.32 (0.06)	2.43 (0.08)
h^2^	0.36 (0.06)	0.15 (0.08)	0.48 (0.05)	0.67 (0.03)	0.65 (0.04)	0.71 (0.04)

The genetic variances (σ_a_^2^), error variance (σ_e_^2^), and narrow-sense heritabilities (*h*^2^) were calculated for the entire population and individual families.

^a^Standard error.

**Table 5:** Accuracy of GEBVs assessed by five-fold cross-validation based on a combined dataset consisting of five scallop families.

	*r*(*y*, *EBV*)			*r*(*TBV*, *EBV*)^b^		
	SH	SL	SW	SH	SL	SW
LASSO	0.20 (0.09)^a^	0.27(0.13)	0.15 (0.10)	0.29 (0.13)	0.39 (0.19)	0.25 (0.17)
RR-BLUP	0.30 (0.16)	0.37 (0.09)	0.18 (0.08)	0.43 (0.23)	0.53 (0.13)	0.30 (0.13)
BL	0.31 (0.16)	0.36 (0.08)	0.15 (0.07)	0.44 (0.23)	0.51 (0.12)	0.25 (0.12)
G-BLUP	0.37 (0.08)	0.32 (0.09)	0.33 (0.09)	0.53 (0.12)	0.46 (0.13)	0.55 (0.15)
BayesA	0.40 (0.07)	0.33 (0.08)	0.35 (0.09)	0.57 (0.10)	0.47 (0.12)	0.58 (0.15)
BayesB	0.40 (0.07)	0.34 (0.07)	0.36 (0.08)	0.57 (0.10)	0.49 (0.10)	0.60 (0.13)

^a^Standard error.

^b^The correlation between EBV and TBV is calculated as the *r*(*y*, *EBV*) divided by the square root of the heritability of a given trait.

**Table 6:** The correlation of marker effects estimated using five GS models based on a combined family dataset for the trait of shell length.

	LASSO	RR-BLUP	BL	BayesA	BayesB
LASSO	1.00	0.32	0.30	0.26	0.23
RR-BLUP		1.00	0.82	0.72	0.72
BL			1.00	0.62	0.62
BayesA				1.00	0.87
BayesB					1.00
