# Supplementary material for: Evaluation of the 2b-RAD method for genomic selection in scallop breeding
Source: Sci Rep. 2016 Jan 12;6:19244. doi: 10.1038/srep19244 (PMC4709697; doi:10.1038/srep19244)

Evaluation of the 2b-RAD method for genomic selection in scallop breeding

Jinzhuang Dou, Xue Li, Qiang Fu, Wenqian Jiao, Yangping Li, Tianqi Li, Yangfan Wang, Xiaoli Hu, Shi Wang, & Zhenmin Bao**

Supplementary Figures

Figure S1. Principal component analysis (a) and genetic kinships (b) of the simulated 20 full-sib families using the markers derived from the low-density panel (2,364). The numbers in brackets refer to the proportion of variance explained.

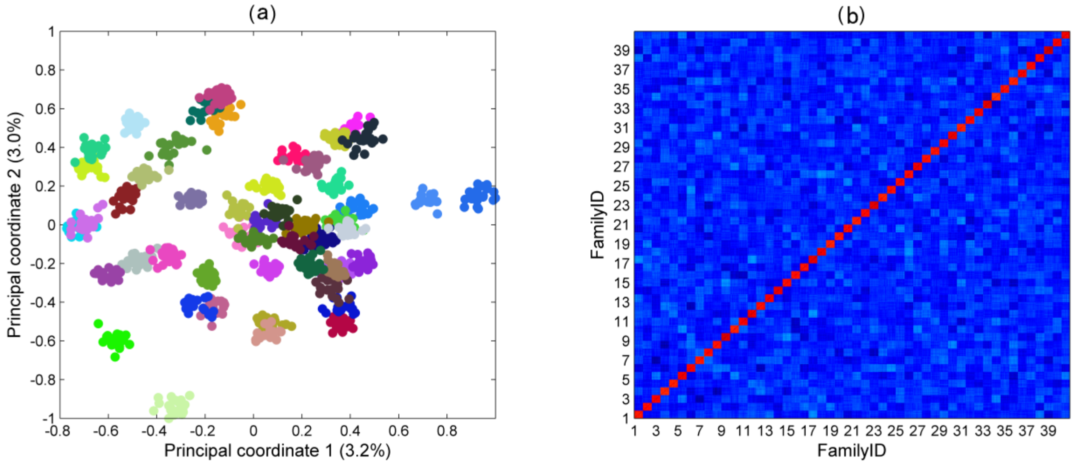

Figure S2. Accuracy of GEBVs estimated from a family-based dataset considering the dominant effects (Simulation 3). The vertical bar indicates the prediction accuracy under each scenario. The x-axis indicates the proportion of explained variation of additive effects, whereas the y-axis indicates the proportion of explained variation of dominant effects.

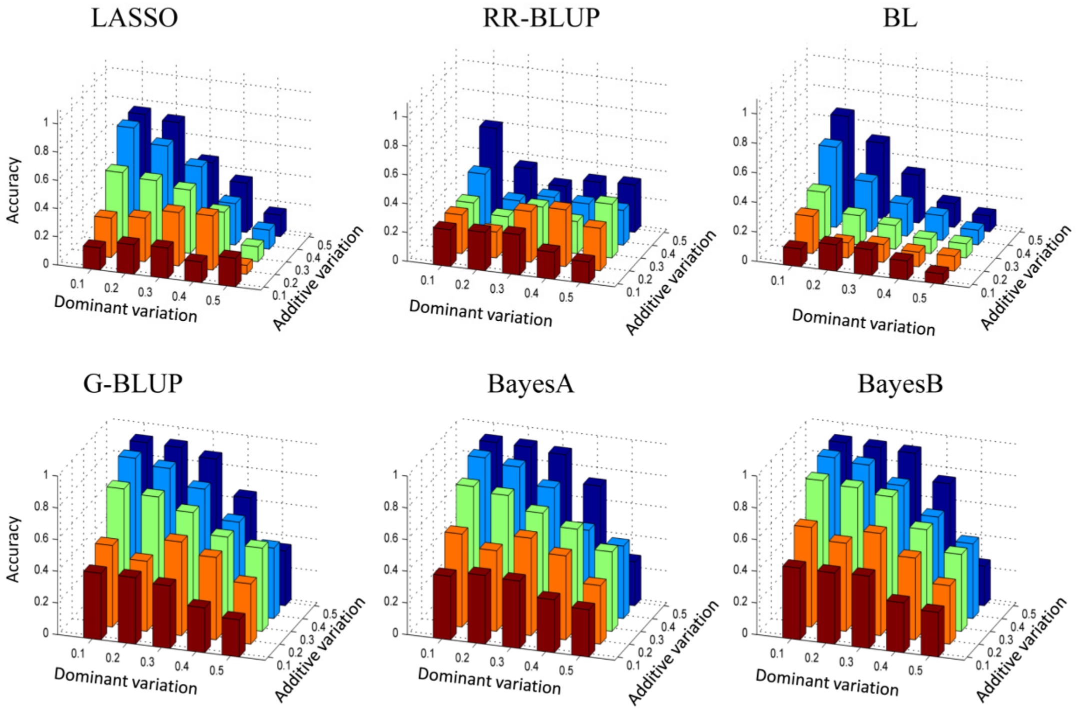

Supplement: Supplementary Figures S1 and S2 [file srep19244-s1.pdf]
